# Vibration and β‐hydroxy‐β‐methylbutyrate treatment suppresses intramuscular fat infiltration and adipogenic differentiation in sarcopenic mice

**DOI:** 10.1002/jcsm.12535

**Published:** 2020-01-28

**Authors:** Jinyu Wang, Can Cui, Yu Ning Chim, Hao Yao, Liu Shi, Jiankun Xu, Jiali Wang, Ronald Man Yeung Wong, Kwok‐Sui Leung, Simon Kwoon‐Ho Chow, Wing Hoi Cheung

**Affiliations:** ^1^ Musculoskeletal Research Laboratory, Department of Orthopaedics and Traumatology The Chinese University of Hong Kong Hong Kong SAR The People's Republic of China; ^2^ The CUHK-ACC Space Medicine Centre on Health Maintenance of Musculoskeletal System The Chinese University of Hong Kong Shenzhen Research Institute Shenzhen The People's Republic of China

**Keywords:** Fat infiltration, HMB, LMHFV, MDSC, Sarcopenia, Wnt/β‐catenin

## Abstract

**Background:**

Sarcopenia is an aging‐induced deterioration of skeletal muscle mass and function. Low‐magnitude high‐frequency vibration (LMHFV) was shown to improve muscle functions and β‐hydroxy‐β‐methylbutyrate (HMB) to increase muscle mass and strength. Muscle‐derived stem cells (MDSCs) are progenitor cells important for muscle regeneration. We hypothesized that LMHFV and HMB could retard sarcopenia by reducing fat infiltration through inhibiting adipogenesis in MDSCs.

**Methods:**

Senescence‐accelerated mouse P8 male mice were randomized into control (CTL), HMB, LMHFV (VIB), and combined (COM) groups. Interventions started at age of month 7 and assessed at 1, 2, and 3 months post‐intervention by densitometry, histology, and functional tests. *In vitro*, MDSCs isolated from gastrocnemius of senescence‐accelerated mouse P8 mice were characterized, randomized into CTL, VIB, HMB, and COM groups, and assessed by oil red O staining, mRNA, and protein expression.

**Results:**

At 2 months post‐intervention, percentage lean mass of HMB, VIB, and COM groups were significantly higher than CTL group. Twitch, tetanic, and specific tetanic forces of COM group were higher, while specific twitch force of both VIB and COM groups were higher. Grip strength of HMB, VIB, and COM groups were higher. Histologically, both VIB and COM groups presented lower oil red O area than CTL group. Type I muscle fibre in CTL group was higher than HMB, VIB, and COM groups. MDSC were detected *in situ* by immunofluorescence stain with stem cell antigen‐1 signals confirmed with higher β‐catenin expression in the COM group. The observations were also confirmed *in vitro*, MDSCs in the HMB, VIB, and COM groups presented lower adipogenesis vs. the CTL group. β‐Catenin mRNA and protein expressions were lower in the CTL group while their relationship was further validated through β‐catenin knock‐down approach.

**Conclusions:**

Our results showed that combined LMHFV and HMB interventions enhanced muscle strength and decreased percentage fat mass and intramuscular fat infiltration as compared with either treatment alone. Additive effect of LMHFV and HMB was demonstrated in β‐catenin expression than either treatment in MDSCs and altered cell fate from adipogenesis to myogenesis, leading to inhibition of intramuscular lipid accumulation. Wnt/β‐catenin signalling pathway was found to be the predominant regulatory mechanism through which LMHFV and HMB combined treatment suppressed MDSCs adipogenesis.

## Introduction

Sarcopenia is defined as ‘a syndrome characterized by progressive and generalized loss of skeletal muscle mass and strength with a risk of adverse outcomes’^1^. The prevalence of sarcopenia based on the definition of the European Working Group on Sarcopenia in Older People was reported 1–29% for community‐dwelling elderly people.^2^ The cause of sarcopenia is multifactorial including inactivity, neuromuscular junction degeneration, hormone or cytokine imbalance, and inadequate nutrition.^3^ Aging is associated with changes in both total and regional fat distribution where adipose tissue plays an important role in muscle physiological and pathological processes. Intermuscular adipose tissue accumulation increased with aging and was associated with muscle dysfunction and obesity.^4^

Low‐magnitude high‐frequency vibration (LMHFV) is a type of mechanical stimulation with magnitude lower than 1.0 g (gravitational acceleration).^5^ There are many clinical^6^, ^7^ and preclinical^8^, ^9^ evidences confirming the positive effects of vibration treatment on enhancing muscle performance in elderly and reducing intramuscular lipids, respectively.

β‐Hydroxy β‐methylbutyrate (HMB) is a leucine metabolite produced in tissues of humans and animals, which has positive effects on lean muscle mass and strength when applied together with resistance training or creatine supplement.^10^, ^11^, ^12^ HMB supplementation was also reported to increase fat free mass and reduce fat mass in trained athletes and hence improved body composition and muscle strength.^13^

Muscle‐derived stem cells (MDSCs), which may represent a predecessor of satellite cells, possess the ability to differentiate into other mesodermal cell types and are of high myogenic capacity.^14^, ^15^ Deasy *et al*. reported that the transplantation of vascular endothelial growth factor‐expressing MDSCs could help to repair skeletal muscle damage by modulating angiogenesis, regeneration, and fibrosis in the dystrophic skeletal muscle.^16^ Therefore, MDSCs are of intriguing potential in muscle regeneration, which need further experiments to investigate.

Based on the aforementioned evidences, the hypotheses of the present study were (i) the co‐application of LMHFV and HMB could retard sarcopenia through reducing fat infiltration in sarcopenic senescence‐accelerated mouse P8 (SAMP8) mice model and (ii) LMHFV and HMB co‐application could suppress adipogenic activity of MDSCs through regulating putative signalling pathways *in vitro*.

## Materials and methods

### Animal model

Senescence‐accelerated mouse P8 mice characterized to exhibit sarcopenia onset at month 8^17^ was used in this study. Male mice were used to avoid hormone variation. A total of 96 SAMP8 male mice were randomized into four groups: control (CTL), HMB only (HMB), LMHFV treatment only (VIB), and combined treatment (COM) group. A separate batch of 12 mice (*n* = 3 per group) were used for the *in situ* detection of MDSC and β‐catenin expression. Briefly, interventions started at the age of month 7 and the mice were harvested at three time points (1, 2, and 3 months post‐intervention; *n* = 8 per group per time point). In HMB group, HMB supplement (500 mg/kg/day, 5 days/week) was given to mice.^18^ In VIB group, mice were given LMHFV treatment (35 Hz, 0.3 g, 20 min/day, 5 days/week) according to our previous protocol.^8^ In COM group, both LMHFV and HMB supplement were provided accordingly. The mice in CTL group were put on vibration platform with power off to receive sham treatment with the same regime. All of the mice were euthanized at specified time points for the following assessments. The research protocol was approved by the Animal Experimentation Ethics Committee of the Chinese University of Hong Kong (Ref: 15‐200‐MIS).

At endpoints, body mass of mice was measured before euthanasia.^8^, ^17^ Gastrocnemii of right hind limbs were carefully isolated with the Achilles tendon and femur condylar attached, which was then put in the tissue bath of mammalian Ringer solution for *ex vivo* functional test. The contralateral muscle was also harvested after weighing, snap frozen with 2‐methylbutane in optimal length for 20 s, and stored at −80°C for histological and immunofluorescence examination. Serum samples were collected and stored at −80°C for myostatin and adiponectin quantification by enzyme‐linked immunosorbent assay (R&D Systems, Minneapolis, MN, USA) as previously reported.^8^

### Grip strength measurement

Forelimb grip strength of mice was measured with a force gauge (Mark‐10 Corporation, NY, USA). Mice held by the tail grasped the grid connected to the force gauge with their fore paws. The tails of mice were pulled slowly until the mice released their fore paws from the grid.^19^, ^20^ The peak force of each test was recorded; three repeats were collected and averaged.

### Dual‐energy X‐ray absorptiometry analysis

Measurement of whole‐body composition was performed using dual‐energy X‐ray absorptiometry (DXA) (UltraFocus DXA, Faxitron, AZ, USA) at specified time points before euthanasia. Under general anaesthesia, mice were placed in the DXA device in prone position with four limbs fixed for scanning. Percentage lean/fat mass were analysed using default BiopticsVision software.

### *Ex vivo* functional test

*Ex vivo* muscle functional test was conducted according to our established protocols using *ex vivo* muscle functional test system (800A, Aurora Scientific Inc., Newmarket, Canada).^8^, ^17^, ^21^ After 15 min stabilization, the muscle was activated by two tetanic contractions (1A, 300 ms, 150 Hz) with 5 min interval. The optimal length (L0) of the muscle was determined by continuous stimulations with increasing muscle length until the new response value was equal to the former one. Twitch force (F0) output was measured at the optimal length. Muscle was stimulated by one electronic stimulus with 1 min interval and the response was defined as F0. Three twitch responses were measured and averaged as the isometric F0. The tetanic force (Ft) was detected by stimulation at 80 Hz for 300 ms. Three tetanic responses were measured at 5 min intervals and averaged as the Ft. After the measurements, the gastrocnemius was dried and weighted. The gastrocnemius from the other leg was also isolated and weighted. Muscle mass was the average of both legs. Muscle cross‐sectional area, specific twitch force (SF0), and specific tetanic force (SFt) were calculated as previously described.^22^

### Micro‐computed tomography

Micro‐computed tomography was performed at the distal side of femur harvested from SAMP8 mice (vivaCT, Scanco Medical, Brüttisellen, Switzerland) based on our protocol.^21^ Images were acquired at 70 kVp and 114 mA, and the region of interest covered 30 slides from the end of growth plates to the proximal direction. Tissues within the threshold range of 220–1000 were segmented, reconstructed, and evaluated for the volume of mineralized bone.

### Histological and immunofluorescence analysis

Muscle samples collected from the gastrocnemius were cryo‐sectioned for histological examination. Muscle samples sectioned at 8 μm thick (Cryostar NX70, Thermo Scientific, MA, USA) were mounted on silane‐coated glass slides^8^, ^17^ and subjected to H&E and oil red O (ORO) staining for evaluation of intramuscular fat infiltration. Immunofluorescence staining of myosin heavy chain (MHC) was performed for muscle fibre typing based on Bloemberg *et al*.'s protocol.^23^ Primary antibodies against MHCI (BA‐F8), MHC IIa (SC‐71), and MHC IIb (BF‐F3) (DSHB, IA, USA) were mixed to form the primary antibody cocktail. Secondary antibodies including Alexa Fluor 350 IgG2b, Alexa Fluor 488 IgG1, and Alexa Fluor 555 IgM (Invitrogen, CA, USA) were diluted and mixed to form the secondary antibody cocktail. Concentration of both cocktails were 4 μg/mL. After image acquisition (DM6000B, Leica, Wetzlar, Germany), areas of MHC IIa (green), MHC IIb (red), and MHC I (blue) were quantified by Image J software (NIH, Bethesda, USA). For the *in situ* detection of MDSC, primary antibodies of stem cell antigen‐1 (Invitrogen) and laminin (Abcam, USA) were conjugated with Alexa Fluor 488 (green) and 546 (red), respectively.

### Western blot analysis

Muscle samples from the *in vivo* study were harvested and digested in lysis buffer with complete mini protease/phosphatase inhibitor cocktail (Cell Signaling Technology, USA) according to instruction by the manufacturer. Harvested cells for *in vitro* studies were lysed using radioimmunoprecipitation assay buffer with complete mini protease inhibitor cocktail (Roche, USA) and soluble protein was collected by centrifugation at 18 407 rcf for 10 min at 4°C. Western blot analysis was performed according to previous protocol.^24^ Antibodies used were as follows: β‐catenin (1:3000; Cell Signaling Technology) and β‐actin (1:4000; Sigma, USA). Results were visualized on the GeneGnome XRQ (Syngene, Cambridge, UK) for the *in vivo* study or X‐ray film (Fujifilm, Japan) for the *in vitro* study.

### Isolation of muscle‐derived stem cells

To further study the mechanism of LMHFV and HMB on the fat metabolism *in vitro*, MDSCs were isolated from muscle tissues. MDSCs were isolated following Gharaibeh *et al*.'s protocol.^25^ Gastrocnemii of SAMP8 mouse were isolated, minced, and washed with Hank's Balanced Salt Solution (Invitrogen). It was re‐suspended in 10 mL of 0.2% collagenase‐type XI (Sigma) and incubated in a 5% CO_2_ incubator for 1 h at 37°C. The mixture was then centrifuged, and supernatant was removed and incubated in 10 mL of dispose solution in 5% CO_2_ incubator at 37°C for 45 min. The mixture was centrifuged again, supernatant removed and incubated in 10 mL 0.05% Trypsin‐PBS (Invitrogen) for 30 min at 37°C, centrifuged again and re‐suspended in proliferation media [DMEM, 10% FBS, 10% (vol/vol) horse serum (Invitrogen), 0.5% (vol/vol) chick embryo extract (USbio, USA)]. The cell resuspension was passed through a cell strainer (BD Biosciences, CA, USA) and the passed cells were put in a collagen‐coated T‐25 flask (Corning, NY, USA) marked as PP1 and incubated in 5% CO_2_ incubator at 37°C for 2 h. The floating cells in the supernatant were transferred to another flask and incubated in 5% CO_2_ incubator at 37°C for 24 h, marked as PP2. The manipulation was repeated every 24 h until PP6 was created. The cells were viewed as slow adhering cells that contained MDSCs for further application.

### Muscle‐derived stem cell multi‐lineage differentiation induction

Muscle‐derived stem cells were seeded in six‐well plates at 2 × 10^5^ cells per well. To perform the multi‐lineage differentiation induction, the cell culture medium was changed to adipogenic induction medium, osteogenic induction medium, or myogenic induction medium during 80% cell confluence. Adipogenic induction medium consists of DMEM supplemented with 10% FBS, 1 μM dexamethasone, 0.2 mM indomethacin, 10 μg/mL insulin, and 0.5 mM 3‐isobutyl‐1‐methilxantin. Osteogenic induction medium contains DMEM supplemented with 10% FBS, 50 μg/mL ascorbic acid, 10 mM β‐glycerophosphate, and 10 nM dexamethasone.^26^ Myogenic induction medium is composed of DMEM with 2% horse serum (Invitrogen). Osteogenic differentiation was evaluated by alizarin red S staining 10 days after induction.^27^ Adipogenic differentiation of MDSCs was evaluated by real‐time PCR and ORO staining 10 days after induction. Myogenic differentiation was evaluated by immunofluorescence staining of MHC IIa 5 days after induction.

### Cell culture and grouping

To further investigate the role of MDSCs in fat infiltration, isolated MDSCs were subjected to adipogenic differentiation and divided into four groups including control (CTL), HMB only (HMB), vibration only (VIB), and combined group (COM), where the HMB group received 50 μM of HMB, the VIB group received 20 min/day of vibration treatment, and the COM group received both. Treatments started when the cells reached around 70% confluence in six‐well plates each containing 1 × 10^5^ cells with interventions lasted for 10 days before assessments. Each experiment was repeated three times.

To perform the RNA silencing, MDSCs were seeded in six‐well plates at 2.5 × 10^4^ cells/well. Cells were grown to 70% confluence and serum‐starved for 24 h. The cells were transfected with either 40 pmol of si‐β‐catenin or scrambled negative control si‐NC in corresponding groups, where si‐β‐catenin (targeting 5′‐GGGUUCCGAUGAUAUAAAUTT‐3′, 5′‐AUUUAUAUCAUCGGAACCCTT‐3′) and si‐NC (targeting 5′‐UUCUCCGAACGUGUCACGUTT‐3′, 5′‐ACGUGACACGUUCGGAGAATT‐3′) (GenePharma Biotechnology, Shanghai, China) were transfected to the MDSCs according to the protocol of Lipofectamine 3000 (Thermo Fisher, USA). The cells were subjected to interventions for 2 days before assessments.

### RNA extraction and real‐time PCR

Total RNA of MDSCs was harvested with RNAiso Plus reagent (TaKaRa, Japan) following the manufacturer's protocol. After extraction, cDNAs were reverse transcribed from RNA using PrimeScript RT Master Mix kit (TaKaRa). Power SYBR Green PCR Master Mix (Thermo Fisher) was used to perform the qRT‐PCR of target mRNA detection. The qRT‐PCR reaction was performed in ABI 7300 Fast Real‐Time PCR Systems (Applied Biosystem, USA) based on our protocol.^28^ The relative fold changes of candidate genes were analysed by using the 2^−ΔΔCt^ method. The primers for real‐time PCR were included in Table 1.

**Table 1:** Primers for real‐time PCR (F: Forward; R: Reverse)

Gene		Sequence	Product size
PPARγ	F	5′‐GGAAGACCACTCGCATTCCTT‐3′	121
	R	5′‐GTAATCAGCAACCATTGGGTCA‐3′	
PGC1α	F	5′‐TATGGAGTGACATAGAGTGTGCT‐3′	143
	R	5′‐GTCGCTACACCACTTCAATCC‐3′	
C/EBPα	F	5′‐GCGGGAACGCAACAACATC‐3′	97
	R	5′‐GTCACTGGTCAACTCCAGCAC‐3′	
β‐catenin	F	5′‐CAGCCGTCAGTGCAGGAG‐3′	129
	R	5′‐CAGCTTGAGTAGCCATTGTCC‐3′	

### Statistical analysis

All quantitative data were expressed as mean ± standard deviation. Data were analysed by one‐way analysis of variance with post hoc Tukey tests using SPSS (SPSS Inc, IBM, USA) for comparison among groups at the same time point. Statistical significance was considered at *P* < 0.05.

## Results

### All treatments enhanced whole‐body composition and reduced fat to lean tissue ratio at the systemic level

**Figure 1:**
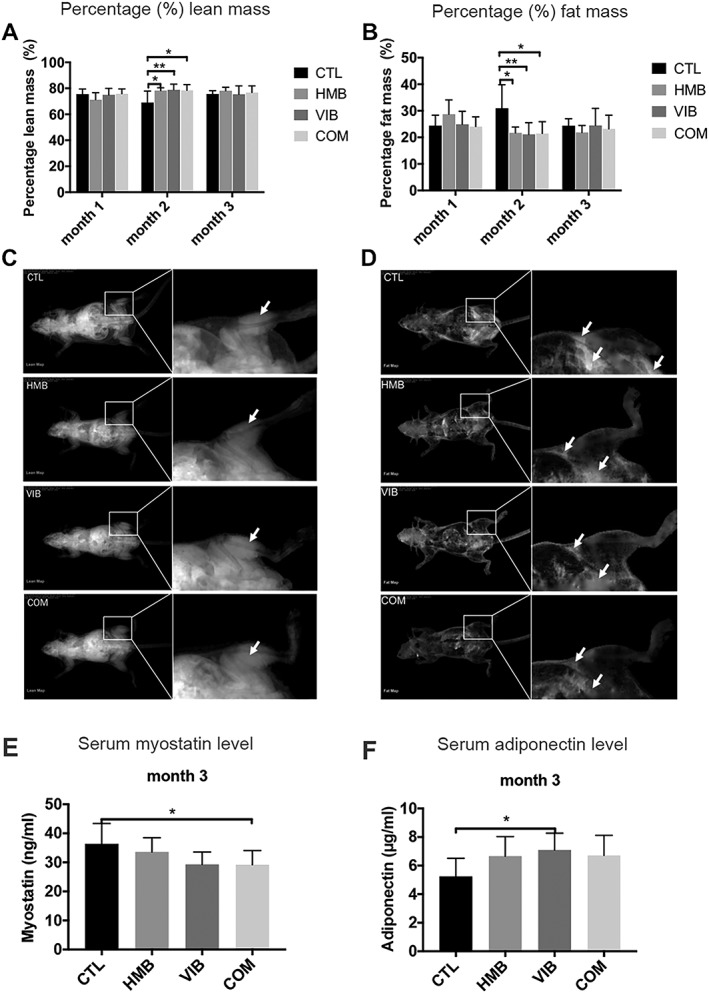
Body composition evaluation of SAMP8 mice by dual energy X‐ray absorptiometry (DXA). (A) Percentage lean mass and (B) percentage fat mass of SAMP8 mice in different groups at 1, 2, and 3 months. Percentage lean mass in CTL group was lower than HMB, VIB, and COM groups at month 2 post‐intervention, while the percentage fat mass in CTL group was higher than HMB, VIB, and COM groups at month 2. (C) Representative image of whole‐body DXA scanning (lean mass mode) (D) and (fat mass mode) demonstrating that the HMB, VIB, and COM groups showed higher percentage lean mass and lower fat mass at month 2, respectively. Lean and fat masses indicated by white arrows. *P < 0.05; **P < 0.01. (E) Serum myostatin level of COM group showed significantly higher than CTL group at month 3. (F) Serum adiponectin level of VIB group showed significantly higher than CTL group at month 3. *P < 0.05. HMB, β‐hydroxy‐β‐methylbutyrate; SAM, senescence‐accelerated mouse.

Treatment groups of HMB, VIB, and COM showed higher percentage lean mass vs. CTL group with significant difference at month 2 as shown by the whole‐body DXA evaluation. No significant difference among groups was found at months 1 and 3 (*Figure* 1A and 1C). For percentage fat mass, HMB, VIB, and COM groups were significantly lower than CTL (*Figure* 1B and 1D) at month 2 and no significant difference was detected at months 1 and 3. Representative DXA images in lean and fat mass modes (*Figure* 1C to 1D) also illustrated that HMB, VIB, and COM treatment groups had higher percentage lean mass and lower fat mass at month 2. No statistical significance in body mass was detected among groups at different time points. Serum biochemistry showed that myostatin level in the COM group was significantly lower than that in the CTL group at month 3 (*P* < 0.05, *Figure* 1E). Adiponectin level in the VIB group was significantly higher than the CTL group at month 3 (*Figure* 1F).

### Combined treatment yielded better treatment effects in intramuscular fat infiltration and fibre typing in sarcopenic skeletal muscle at the tissue level

**Figure 2:**
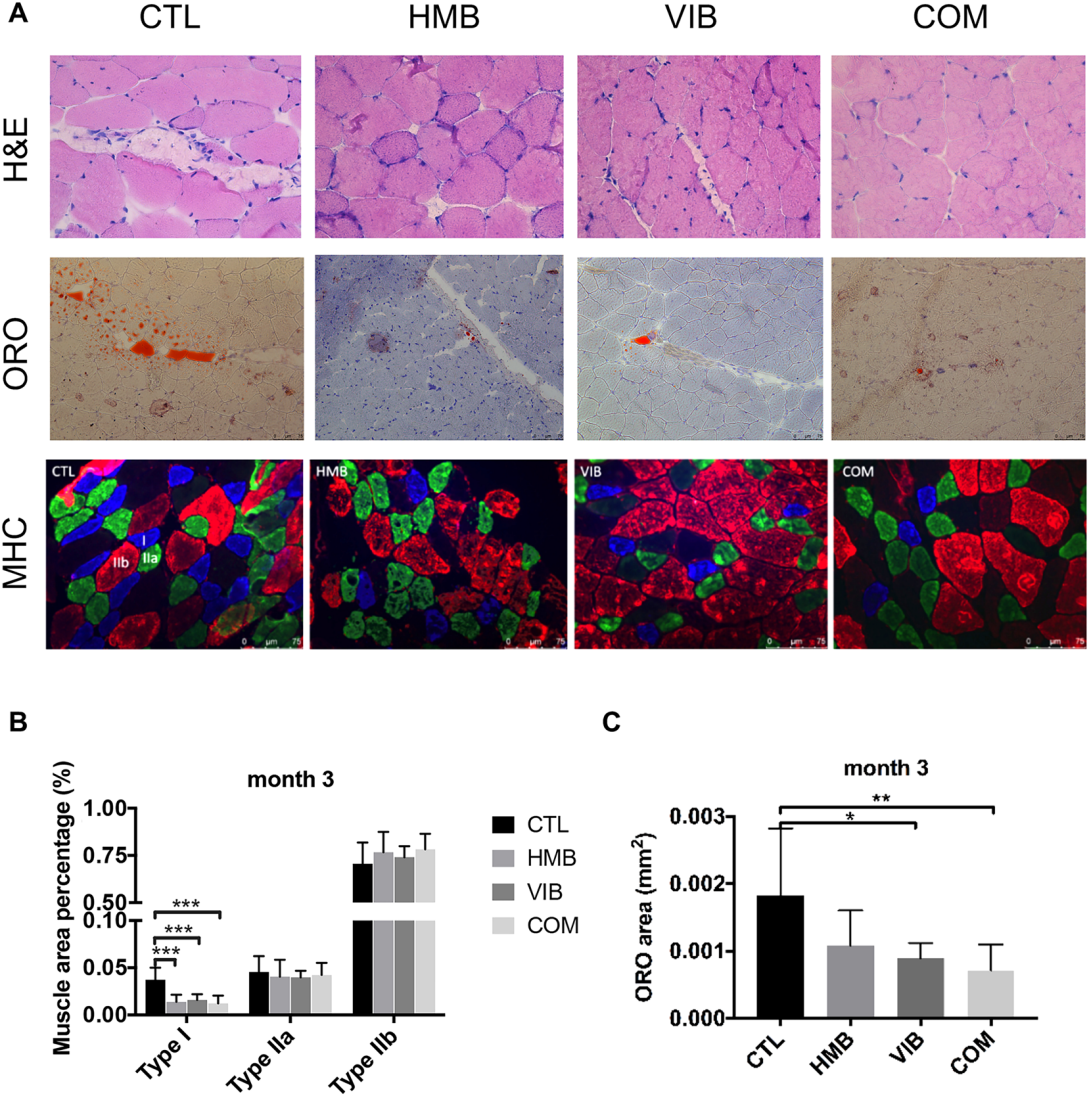
Morphological differences and fat infiltration of skeletal muscles in senescence‐accelerated mouse P8 mice upon different treatments. (A) H&E, oil red O (ORO), and myosin heavy chain (MHC) staining of muscle histology in different groups at month 3. CTL group presented more intramuscular fat tissue than HMB, VIB, and COM groups at month 3 by H&E where fat tissues are indicated by yellow arrows. HMB, VIB, and COM groups presented lower ORO signal (red area) than the CTL group at month 3. At the gastrocnemius, percentage area of type I fibre (blue) in the CTL group was significantly higher than all treatment groups with no significant difference detected in type IIa and IIb fibres at month 3. (B) CTL group showed higher percentage type I fibres than HMB, VIB, and COM groups by MHC immunofluorescence staining at month 3 (***P < 0.001). (C) Quantitative analysis revealed that ORO area of both VIB and COM groups was significantly higher than CTL group at month 3, *P < 0.05, **P < 0.01. HMB, β‐hydroxy‐β‐methylbutyrate.

Various morphological changes were observed by histology of the muscles. Histology showed that both VIB and COM groups presented significantly lower lipid contents at month 3 by 31.07% and 42.58%, respectively, confirmed by ORO staining. The CTL group was also shown to present more intramuscular fat tissue than HMB, VIB, and COM groups at month 3 confirmed with H&E staining (*Figure* 2A). The CTL group generally showed a higher trend of ORO area than the other groups, yet no significant difference detected at months 1 and 2 (*Figure* 2B). Further observations by MHC immunofluorescence staining showed that morphological changes in type I muscle fibre in the CTL group were significantly higher than the HMB group (62.60%), VIB group (57.21%), and COM group (67.07%) at month 3 (*Figure* 2C), where no significant difference detected among groups at shorter time points.

### *In situ* detection of muscle‐derived stem cells demonstrated enhanced β‐catenin expression

**Figure 3:**
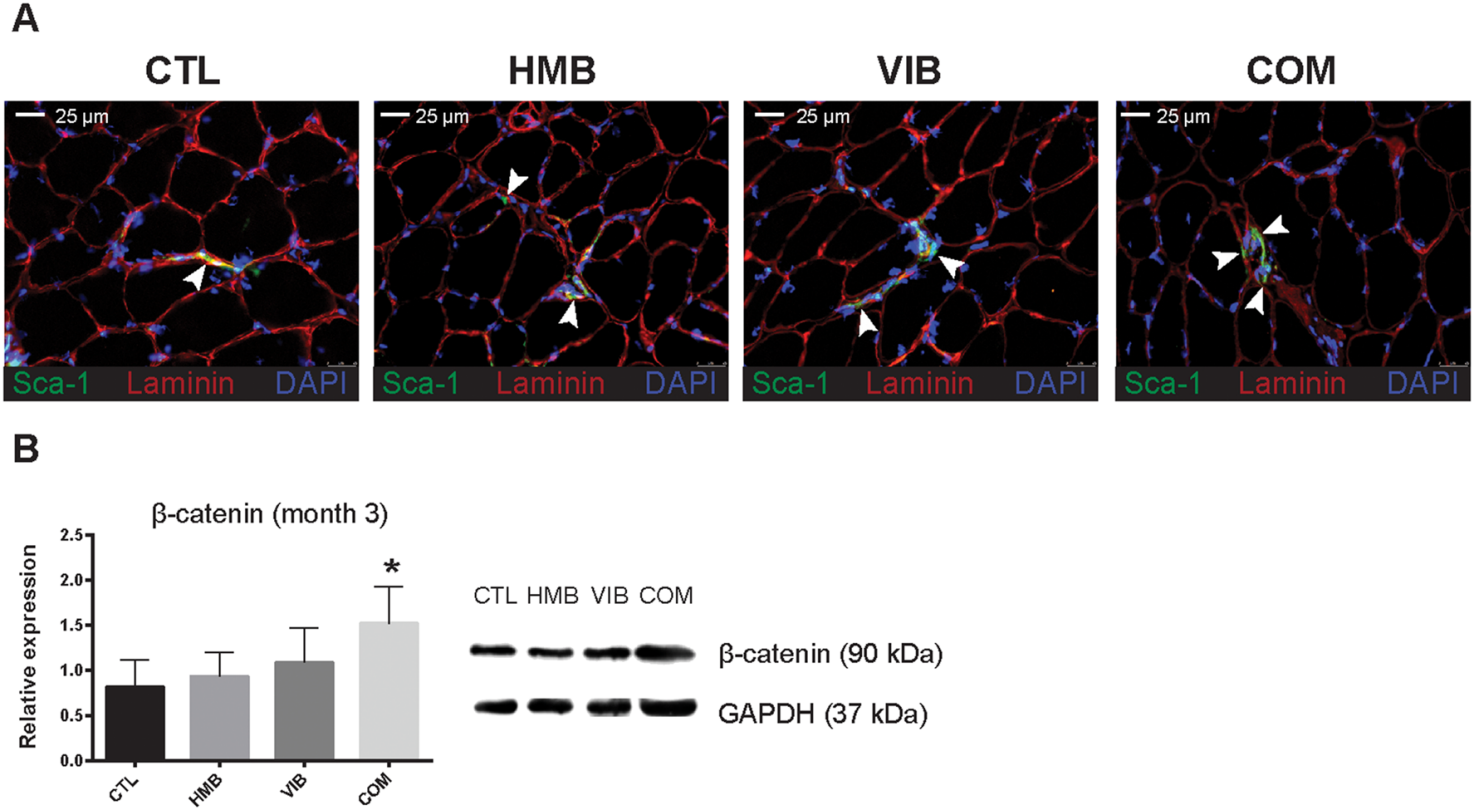
*In situ* detection of muscle‐derived stem cells (MDSC) and β‐catenin expression. (A) MDSC was detected and identified by positive stem cell antigen‐1 (Sca‐1, green) signal, highlighted against basal lamina surrounded muscle fibres (laminin, red) with nuclei stained with DAPI (blue). All MDSC were observed to reside between muscle fibres scattered across the muscle section (white arrows). (B) Western blot analysis showed that total β‐catenin content were increased in the treatment groups compared with the CTL group after 3 months of treatment with only the combine treatment (COM) group reaching statistical significant difference (* *n* = 3, *P* < 0.05, analysis of variance with Bonferroni post hoc test).

Further histological analysis of muscle specimens has been performed to evaluate the potential sources of adipogenesis in the muscle fibres. MDSC identified by stem cell antigen‐1 stem cell markers was identified in all groups at 3 months post‐treatment with generally stronger signals in all treatment groups compared with the CTL group (*Figure* 3A). Furthermore, western blot of β‐catenin also confirmed that the expression of the treatment groups was generally higher in the treatment groups with only the COM group showed statistically significant difference (*P* < 0.05) vs. the CTL suggesting the involvement of β‐catenin in MDSC adipogenic differentiation as observed earlier.

### Combined treatment yielded better enhancements in muscles at the functional level

**Figure 4:**
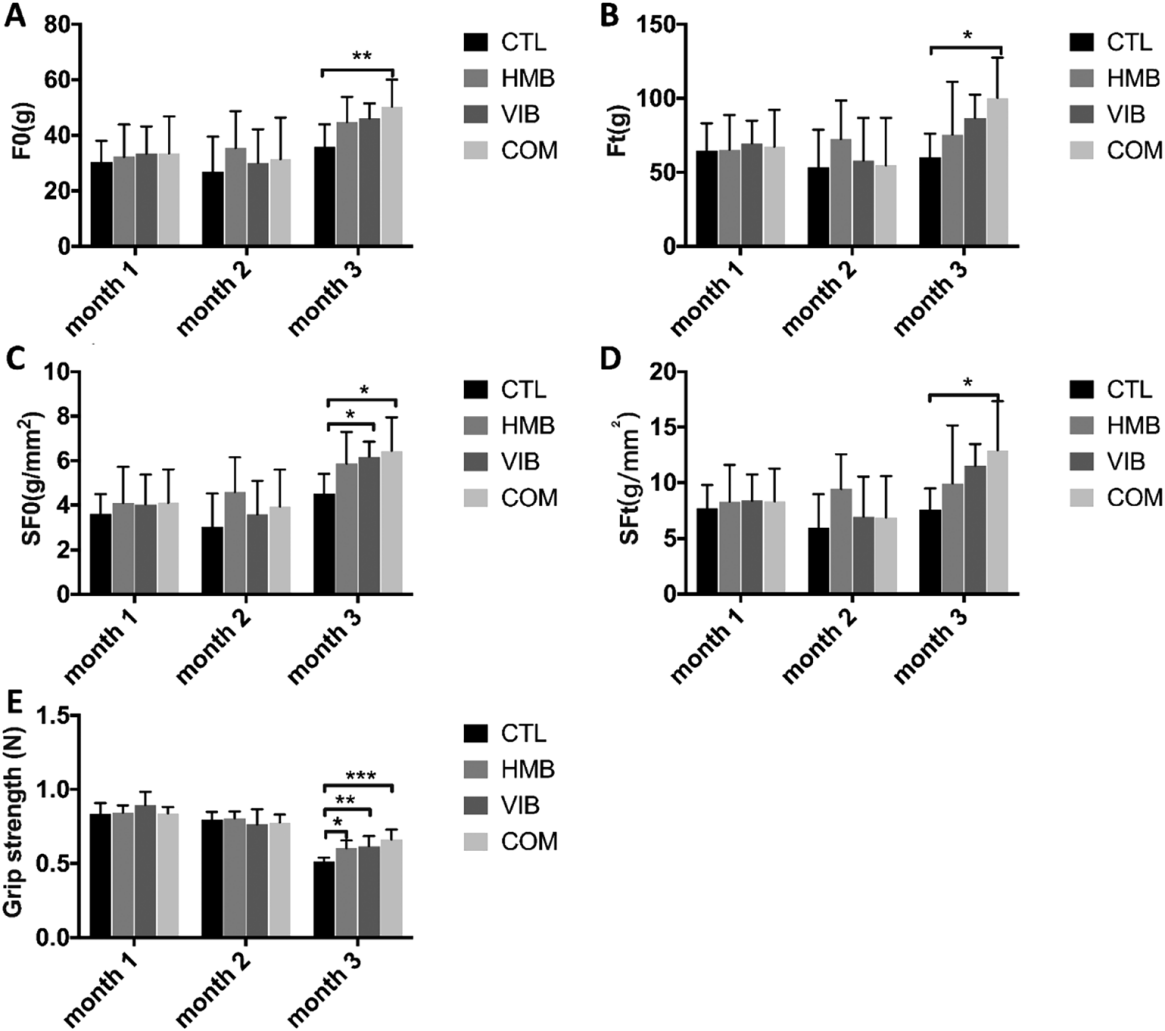
Muscle strength of senescence‐accelerated mouse P8 mice upon different treatments at various time points. (A) Twitch force (F0) of COM group was significantly higher than CTL group at month 3 post‐intervention. No significant difference was found in other groups. (B) Tetanic force (Ft) of COM group was significantly higher than CTL group at month 3 with no significant difference detected in other groups. (C) Specific twitch force (SF0) of VIB and COM groups were significantly higher than CTL group at month 3 with no significant difference detected in other groups. (D) Specific tetanic force (SFt) of COM group was significantly higher than CTL group at month 3 with no significant difference detected in other groups. (E) The grip strength of HMB, VIB, and COM groups were significantly higher than CTL group at month 3. *P < 0.05; **P < 0.01; ***P < 0.001. HMB, β‐hydroxy‐β‐methylbutyrate.

Twitch force of COM group was significantly higher than CTL group at month 3 (*P* < 0.01), while all other groups presented no significant difference. No significant difference in twitch force was detected among groups at months 1 and 2 (*Figure* 4A). A significant increase of tetanic force of COM group than CTL group was found at month 3 (*P* < 0.05), while HMB and VIB groups did not show any significant difference despite their higher trend. No significant difference of tetanic force was seen among groups at months 1 and 2 (*Figure* 4B). Specific twitch force of both VIB and COM groups were significantly higher than CTL group at month 3 (both at *P* < 0.05); HMB group also showed higher specific twitch force, yet without significance. No significant difference among groups was detected at months 1 and 2 (*Figure* 4C). Significantly higher specific tetanic force of COM groups than the CTL group was shown at month 3 (*P* < 0.05). No significant difference was found among groups at months 1 and 2 (*Figure* 4D). Grip strength of HMB, VIB, and COM groups were significantly higher than CTL group at month 3. No significant difference was seen among groups at months 1 and 2 (*Figure* 4E).

### Isolation and identification of muscle‐derived stem cell as a possible source of fat infiltration

Muscle‐derived stem cells were isolated from gastrocnemius, which PP1 cells mainly included the rapidly adhering cells such as fibroblast‐like and myoblast cells. In PP3, slowly adhering cells appeared and looked spherical and refractive. In late pre‐plates (PP6), the slowly adhering cells became typically sparse, which were mainly MDSCs (*Figure* 4A to 4C). For cell characterization, calcium deposits were confirmed by alizarin red S staining after osteogenic differentiation for 10 days; lipid droplets were detected by ORO staining after a 10 day adipogenic induction; and myotube formation by immunofluorescence staining after a 5 day myogenic induction (*Figure* 4D to 4F).

### Inhibition of adipogenic differentiation of muscle‐derived stem cell by vibration, β‐hydroxy‐β‐methylbutyrate, or combined treatment

**Figure 5:**
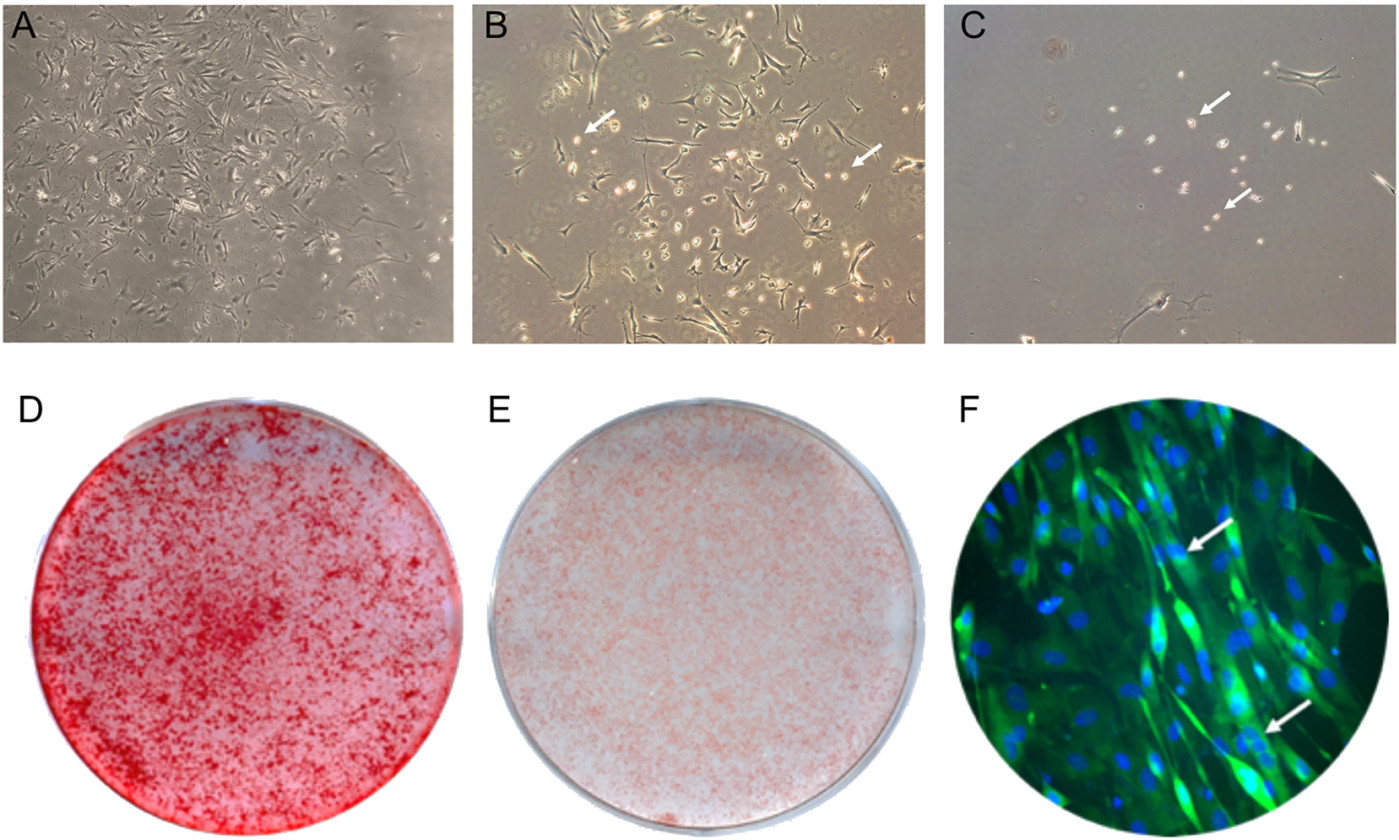
Morphology and characterization of muscle‐derived stem cells (MDSC) at different stages of their isolation utilizing the pre‐plate technique. (A) PP1 cells isolated from muscle of SAMP8 mice. (B) PP3 cells from muscle of SAMP8 mice. (C) PP6 MDSCs isolated from muscle of SAMP8 mice. MDSCs are indicated by yellow arrows. (D) Alizarin red S staining at day 10 after osteogenic induced culture in MDSCs. (E) Oil red O staining at day 10 after adipogenic induced culture in MDSCs. (F) Immunofluorescence staining of MDSCs at day 5 after myogenic induction. Myotubes are indicated by white arrows, green area is myosin heavy chain IIa positive, and the blue area is nucleus stained by DAPI. SAM, senescence‐accelerated mouse.

After 10 day adipogenic induction of MDSCs, the CTL group showed more adipogenic cells than the HMB, VIB, and COM groups. Quantitatively, HMB, VIB, and COM groups presented significantly lower ORO area than CTL group, while COM group had significantly fewer adipogenic cells than HMB group (*P* < 0.01) (*Figure* 5A to 5B). Expression of PPARγ mRNA in the CTL group was significantly higher than the HMB, VIB, and COM groups, while the HMB group also showed significantly lower expression of PPARγ than the VIB and COM groups. For PGC1α expression, the CTL group was significantly higher than both the VIB and COM groups, while the expression in the COM group was significantly lower than the HMB group. For C/EBPα expression, the CTL group was significantly higher than the COM group (*P* < 0.05) and marginally higher than the HMB group (*P* = 0.065) (*Figure* 5C to 5E). Furthermore, relative mRNA expression of β‐catenin in the CTL group was significantly lower than both the HMB and COM groups (both at *P* < 0.05). At the protein level, the HMB, VIB, and COM groups all showed significantly higher β‐catenin expression than the CTL group, while the COM group was higher than both the HMB and VIB groups (both at *P* < 0.05); β‐catenin expression of the VIB group was significantly higher than the HMB group (*P* < 0.05) (*Figure* 5F to 5G).

### Inhibition of adipogenic differentiation in muscle‐derived stem cell was β‐catenin dependent

**Figure 6:**
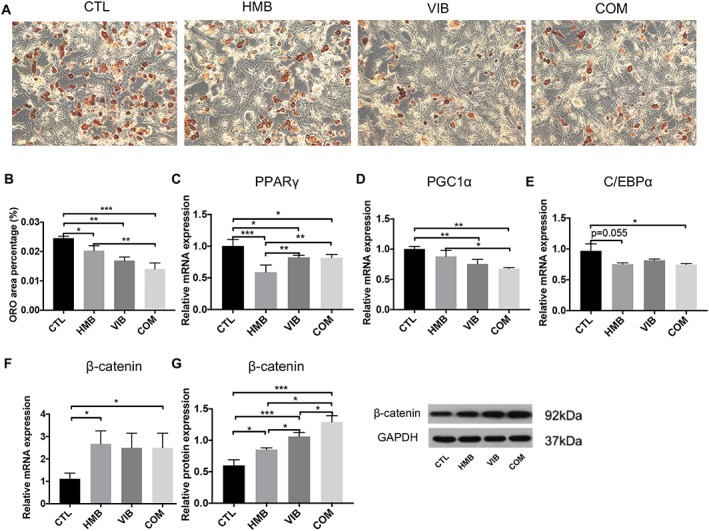
ORO staining of MDSCs after adipogenic induction upon different treatments. (A) CTL group showed more adipogenic cells than HMB, VIB, and COM groups. (B) Quantitative data showed that all treatment groups presented significantly less ORO positive area than the CTL group. The COM group showed significantly less adipogenic cells than HMB group. (C) PPARγ mRNA expression level in CTL group was significantly higher than HMB, VIB, and COM groups. (D) PGC1α mRNA expression in CTL group was significantly higher than VIB and COM groups. PGC1α mRNA expression in COM group was significantly lower than HMB group. (E) C/EBPα mRNA expression in CTL group was significantly higher than COM group and marginally higher than HMB group. (F) Relative expression of β‐catenin mRNA in CTL group was significantly lower than HMB and COM groups. (G) Western blot results revealed that HMB, VIB, and COM groups had significantly higher β‐catenin protein level than CTL group. *P < 0.05; **P < 0.01; ***P < 0.001. HMB, β‐hydroxy‐β‐methylbutyrate; ORO, oil red O.

**Figure 7:**
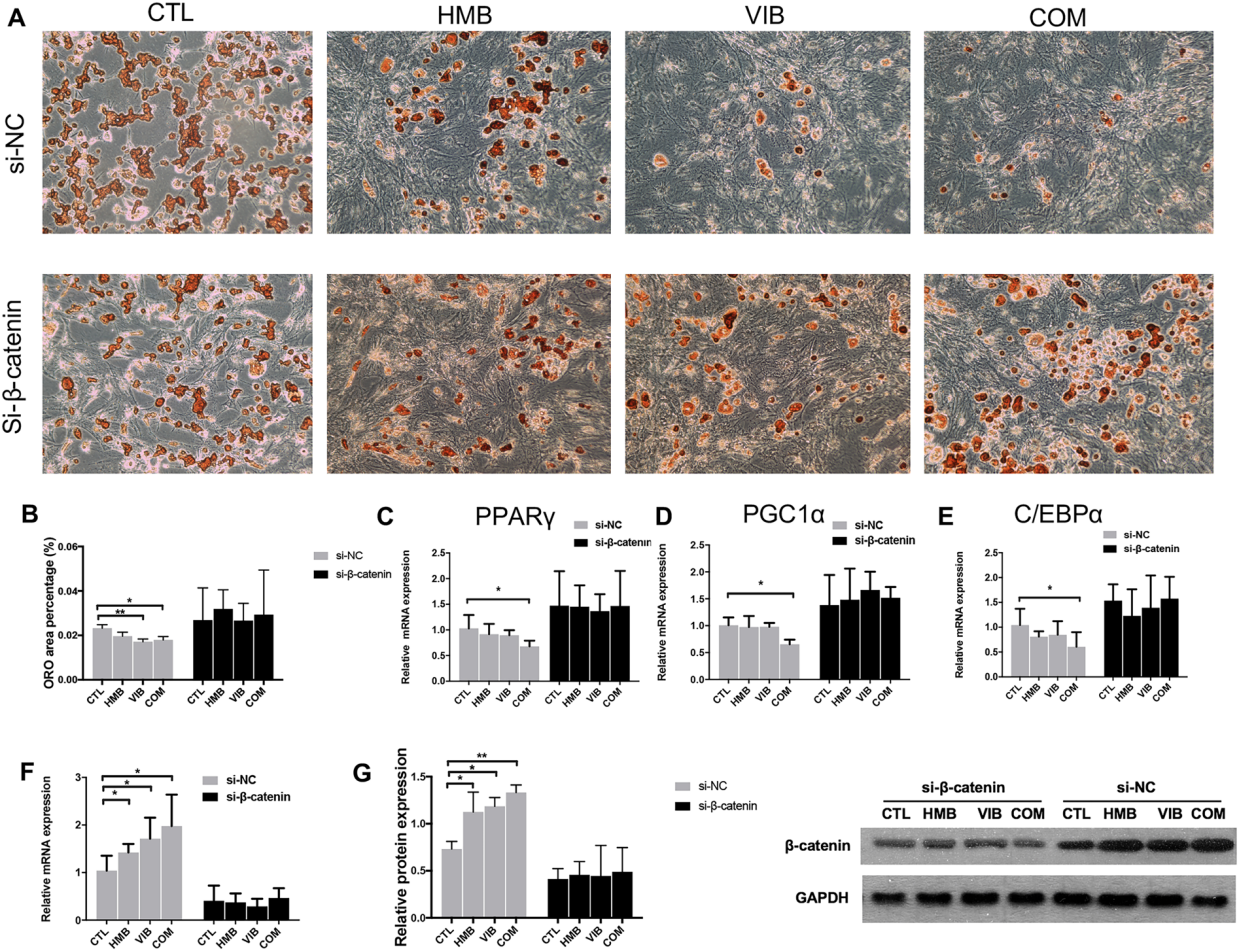
Treatment effects on adipogenesis differentiation in MDSC abolished by si‐β‐catenin knockdown. (A) CTL group showed more adipogenic cells than HMB, VIB, and COM groups after si‐NC knockdown, while no obvious difference was seen among groups after si‐β‐catenin knockdown. (B) Quantitative data showed that VIB and COM groups presented significantly less ORO area than CTL group after si‐NC knockdown, while no significant difference was seen among groups after si‐β‐catenin knockdown. (C) PPARγ mRNA expression in CTL group was significantly higher than COM group after si‐NC knockdown, while no significant difference was seen among groups after si‐β‐catenin knockdown. PGC1α mRNA expression in CTL group was significantly higher than COM group after si‐NC knockdown. No significant difference was seen among groups after si‐β‐catenin knockdown. C/EBPα mRNA expression in CTL group was significantly higher than COM group after si‐NC knockdown. No significant difference of C/EBPα mRNA was seen among groups after si‐β‐catenin knockdown. (F) Relative expression of β‐catenin mRNA in CTL group was significantly lower than HMB, VIB, and COM groups after si‐NC knockdown, but no significant difference was seen among groups after si‐β‐catenin knockdown. (G) Western blot result revealed that no significant difference of β‐catenin protein level was seen among groups after si‐β‐catenin knockdown, while HMB, VIB, and COM groups had significantly higher β‐catenin protein level than CTL group after si‐NC knockdown. *P < 0.05; **P < 0.01. HMB, β‐hydroxy‐β‐methylbutyrate; MDSC, muscle‐derived stem cell; ORO, oil red O.

To investigate the relationship between interventions and Wnt/β‐catenin signalling, β‐catenin silencing approach was used. During adipogenic differentiation, the VIB and COM groups presented significantly less ORO area than the CTL group after negative control si‐NC knockdown, but no significant difference was seen among groups after si‐β‐catenin knockdown (*Figure* 6A to 6B). Consistently, relative expressions of PPARγ, PGC1α, and C/EBPα in CTL group were all significantly higher than COM group (*P* < 0.05) after si‐NC knockdown, but no significant difference was seen among groups after si‐β‐catenin knockdown (*Figure* 6C to 6E). The expressions of β‐catenin in MDSC were enhanced in the HMB, VIB, and COM groups compared with the CTL group with si‐NC knockdown treatment but eliminated by si‐β‐catenin knockdown at both the mRNA and protein levels (*Figure* 6F to 6G) (Figure 7).

## Discussion

This study investigated the co‐application of LMHFV and HMB on sarcopenia through reducing intramuscular fat infiltration and adipogenic activities of MDSCs. In this study, *in vivo* results using comprehensive assessments have revealed that the combined treatment could significantly enhance muscles in terms of quality and performance by suppressing MDSC adipogenesis, through the activation of the β‐catenin signalling pathway. *In vitro*, the combined treatment group also demonstrated similar results with decreased adipogenic differentiation of the MDSC with increased β‐catenin‐related signalling. Taken together, the co‐application of LMHFV and HMB supplementation could effectively retard sarcopenia by reducing fat infiltration and MDSC adipogenesis, which is of intriguing potential to be a new intervention strategy for sarcopenia.

This is the first study to investigate the combined effect of LMHFV and HMB on sarcopenia in animal model. In our previous study, the positive effects of LMHFV alone were demonstrated, with enhanced muscle performance (muscle strength and contractibility), increased muscle fibre type IIA, promoted satellite cell pool, and suppressed myostatin expression. However, muscle mass remained unchanged^8^ hence the aim of this study was to co‐apply LMHFV and HMB to stimulate both muscle mass and performance. Results showed that all muscle performance‐related parameters were significantly enhanced in the combined treatment group at either month 2 or 3 post‐intervention. Some enhancement was only shown in the combined treatment group but not in individual treatment groups (e.g. twitch force, tetanic force, and myostatin expression). In addition to the significantly increased lean muscle mass, all these confirmed the superior capacity of the combined treatment against sarcopenia. As there were some clinical evidences showing the positive effects of HMB supplementation and resistance exercise on muscle mass and performance of adults in randomized controlled trial,^29^ co‐application of LMHFV and HMB may be considered in the future because the compliance of LMHFV for elderly was good with at least 60%^7^, ^30^ that may be an advantage over resistance exercise in clinical applications.

Fat infiltration in aged skeletal muscle detected by computed tomography was previously reported.^31^ The progressive fat accumulation could have many negative impacts on muscle structure, function, and eventually mobility.^32^, ^33^, ^34^ For the effect of the combined treatment on fat infiltration, the results of both percentage fat mass and ORO signals supported the good effect of the combined treatment on decreasing fat infiltration at systematic and tissue levels. To compare with the individual treatment groups (HMB and VIB), COM group was not different in percentage fat mass and was slightly lower than other groups in ORO staining. Hence, COM, HMB, and VIB showed similar effect on reducing fat infiltration *in vivo*, although a higher suppression effect of adipogenesis in COM group over individual treatment groups was found *in vitro*. Detection of MDSC and enhanced β‐catenin expression in muscle histology substantiated with the *in vitro* results indicate that the combined treatment was effective in decreasing adipogenesis in MDSCs, particularly in ORO signals and PGC1α expression. On the other side, very few studies reported the combined effect of HMB supplementation and resistance exercise on adipogenesis, which in Stout's study indicated that the combined treatment could significantly decrease the abdominal fat mass than no‐training group, HMB only group, and resistance exercise only group in 48 heathy elderly men.^35^ These results supported that co‐application of LMHFV and HMB could retard sarcopenia through reducing fat infiltration.

For the individual treatments, LMHFV or HMB demonstrated beneficial effects on enhancing muscle quality and reducing fat infiltration to different extents, despite not as good as the combined treatment group. On muscle parameters, LMHFV could significantly increase percentage lean mass, grip strength, specific twitch force, and decrease muscle fibre type I at either month 2 or 3 post‐intervention. These were substantiated by our previous animal findings^8^ and other clinical evidences in normal community elderly.^7^, ^36^ In recent years, some clinical trials on 12 week vibration treatment (40 Hz × 360 s) for sarcopenia were reported, which demonstrated significant improvement in physical performance (timed‐up‐and‐go test by −15.08%, 10 m walking test by +11.18%, and five‐repetition sit‐to‐stand test by −16.12%) in sarcopenia subjects.^37^, ^38^ Chang *et al*. also reported that 12 week whole‐body vibration (12 Hz, 3 mm) improved the skeletal muscle mass index, physical health, and quality of life in the elderly with sarcopenia.^39^ Among those significantly enhanced muscle parameters in our study, VIB group showed comparable increase as COM group, with most of them higher than HMB group despite no significant difference. Hence, LMHFV should contribute much on muscle quality enhancement in the co‐application intervention, as the COM group showed more pronounced increase and more significantly enhanced parameters (e.g. twitch force, tetanic force, and specific twitch force), representing an additive effects of LMHFV and HMB against sarcopenia. On fat infiltration, LMHFV could also significantly decrease percentage fat mass and lipid contents and increase adiponectin expression at either month 2 or 3 post‐intervention, where adiponection is an adipokine secreted from adipocytes^40^ and the level was lower in sarcopenia individuals than non‐sarcopenia ones.^41^ This was also supported by two studies which reported that vibration (90 Hz, 0.3 g) could reverse the elevated adipogenic gene expression induced by ovariectomy in C57BL/6 mice^42^ and vibration (30 Hz, 1.9 g) partially reversed aging‐induced visceral adiposity in C57BL6/JOlaHsd mice.^43^

On muscle parameters, HMB only group could significantly increase percentage lean mass and grip strength and decrease muscle fibre type I at either month 2 or 3 post‐intervention. Nissen *et al*. also revealed that HMB prevented muscle from degradation (muscle strength increased by 13% at 1.5 g/day HMB and 18% at 3.0 g/day HMB) and even improved muscle function with resistance training.^44^ Despite the improvement, HMB was not as effective as COM or VIB groups in enhancing muscle performance, yet HMB could provide additive effect with LMHFV against sarcopenia. Meanwhile, some clinical evidences challenged that the effect of HMB on muscle performance was not remarkable in adults.^45^ On fat infiltration, HMB could only significantly reduce the percentage fat mass at month 2 post‐intervention, while ORO signal and adiponection remained insignificant. Panton *et al*. reported that percentage fat mass was significantly reduced by 1.1 ± 0.2% in the HMB supplementation group in 75 adults of both genders.^12^ In contrast, Rossi's review conveyed that the role of HMB in sarcopenic obesity management is still controversial.^46^ Therefore, HMB should contribute more on muscle improvement, while its effect on fat reduction is not very notable.

In this study, *in vitro* findings provide insight into the adipogenic mechanism of the co‐application of LMHFV and HMB on sarcopenia. To echo the animal data, *in vitro* data supported that the COM group was most effective to suppress adipogenesis of MDSCs with all parameters significantly reduced. Meanwhile, LMHFV contributed much on suppression of adipogenesis with significant reduction of ORO signals and lower expression of PPARγ and PGC1α, which is the first report of the repressive role of LMHFV on MDSC's adipogenesis. Luu's study also substantiated that low‐magnitude mechanical signals (90 Hz, 0.2 g) could reduce adipogenic differentiation of mesenchymal stem cells with down‐regulation of PPARγ and C/EBPα in an obesity mouse model.^47^ Similar to *in vivo* data, the effect of HMB alone on adipogenesis was not remarkable, with significant reduction in ORO signals and reduced expression of PPARγ only. Furthermore, HMB group was significantly higher than COM group in ORO signal and PGC1α, indicating its less effective suppressive ability on adipogenesis. To our knowledge, there is no any report on the effect of HMB on adipogenesis of precursor cells, and our findings may shed light on the related regulatory mechanism.

On mechanism, Wnt/β‐catenin was examined in this study because this was a predominant extracellular signalling pathway to regulate adipogenesis.^48^ The results both *in vivo* and *in vitro* indicated that the mRNA and protein levels of β‐catenin in MDSCs were up‐regulated by LMHFV, HMB, and combined treatment, where the protein level of COM group was the strongest, followed by VIB and HMB groups. These findings support that the proposed interventions could suppress adipogenesis through activating Wnt/β‐catenin signalling in MDSCs. It is speculated that either of these treatments schemes, or in combination, would activate the Wnt/β‐catenin pathway by stimulating the extracellular Wnt‐frizzled through a β‐catenin‐dependent pathway that stabilizes intracellular β‐catenin in the MDSCs for subsequent nuclear translocation to exert their effects of repressing the transcription of adipogenic factors,^49^ as it is also substantiated by the reduced expression of PPARγ and C/EBPα in this study. The knockdown data strongly support this conclusion, as all the suppressive effects of interventions on MDSCs adipogenesis were abolished by si‐β‐catenin. Akimoto's study also supported our findings that mechanical stretch could inhibit myoblasts to differentiate into adipocytes, which was accompanied with an increase of mRNA expression of Wnt/β‐catenin activator Wnt10b.^50^

The limitations of this study were that the senescence change of skeletal muscle in SAMP8 mice might not be similar to humans, although our previous study had characterized the sarcopenic phenotype of SAMP8 animal model.^17^ Also, mice are quadrupedal but humans are bipedal, suggesting their different standing mode on vibration platform.

In conclusion, co‐application of LMHFV and HMB supplement could significantly improve both muscle mass and muscle function in sarcopenic muscle of SAMP8 animal model through reducing fat infiltration. Wnt/β‐catenin signalling pathway is the predominant regulatory mechanism by which LMHFV and HMB suppressed the adipogenesis of MDSCs. This may be the major mechanism of retarding sarcopenia by the co‐application approach. Co‐application of LMHFV and HMB can be considered as a novel intervention strategy for sarcopenia for coming randomized controlled trial and clinical application.

## Conflict of interest

The authors declare no conflict of interest and certify that they comply with the ethical guidelines for authorship and publishing of the Journal of Cachexia, Sarcopenia, and Muscle.^51^
